# Surveillance of human retroviruses in blood samples from patients with hepatitis B and C in São Paulo, Brazil

**DOI:** 10.1590/0037-8682-0378-2019

**Published:** 2020-02-07

**Authors:** Adele Caterino-de-Araujo, Karoline Rodrigues Campos, Tamirez Villas Boas Petrucci, Rafael Xavier da Silva, Marcílio Figueiredo Lemos, Regina Célia Moreira

**Affiliations:** 1 Laboratório de Pesquisa em HTLV, Centro de Imunologia, Instituto Adolfo Lutz, Coordenadoria de Controle de Doenças, Secretaria de Estado da Saúde de São Paulo, São Paulo, SP, Brasil.; 2 Laboratório de Hepatites, Núcleo de Doenças de Transmissão Sanguínea e Sexual, Centro de Virologia, Instituto Adolfo Lutz, Coordenadoria de Controle de Doenças, Secretaria de Estado da Saúde de São Paulo, São Paulo, SP, Brasil.

**Keywords:** HTLV-1/2, HIV, HBV, HCV, Coinfection, Surveillance

## Abstract

**INTRODUCTION:**

Human retroviruses and the hepatitis B and C viruses (HBV and HCV, respectively) share routes of transmission; thus, coinfections occur and could alter subsequent disease outcomes. A preliminary study on human T-lymphotropic virus types 1 and 2 (HTLV-1/2) in serum samples from HBV- and HCV-infected individuals in São Paulo revealed 1.3% and 5.3% rates of coinfection, respectively. These percentages were of concern since they were detected in HTLV-endemic regions and in high-risk individuals in Brazil. The present study was conducted to extend and confirm these data.

**METHODS:**

HTLV-1/2 and human immunodeficiency virus (HIV) infection status were identified in 1,984 sera for HBV and HCV viral load quantification - 1,290 samples from HBV-infected individuals (53.3% men, mean age: 47.1 years) and 694 samples from HCV-infected individuals (56.3% men, mean age: 50.1 years). HTLV-1/2 antibodies were detected by enzyme immunoassay, followed by western blotting and line immunoassay; HIV infection was detected by enzyme immunoassay.

**RESULTS:**

HTLV-1/-2 infection was detected in 1.9% HBV-infected individuals (0.7% HTLV-1 and 1.2% HTLV-2) and in 4.0% (2.4% HTLV-1 and 1.6% HTLV-2) HCV-infected individuals; HIV infection was detected in 9.2% and 14.5%, respectively. Strong associations with HTLV and HIV, male sex, and older age were found in HBV/HTLV and HCV/HTLV-coinfected individuals (*p*<0.05).

**CONCLUSIONS:**

HTLV-1 and HTLV-2 were confirmed to be prevalent in individuals with HBV and HCV in São Paulo; coinfected individuals deserve further clinical and laboratory investigation.

## INTRODUCTION

Brazil has the largest absolute number of human T-lymphotropic virus types 1- and 2- (HTLV-1 and HTLV-2) infected individuals and, consequently, the largest number of HTLV-associated diseases in Latin America^1^. HTLV-1 is the cause of at least two diseases with high mortality and morbidity: adult T cell leukemia/lymphoma (ATLL) and HTLV-1-associated myelopathy/tropical spastic paraparesis (HAM/TSP)^2^. When HTLV-1 and HTLV-2 are associated with other blood**-**borne and sexually transmitted infections (STIs), such as human immunodeficiency virus (HIV), hepatitis B virus (HBV), and hepatitis C virus (HCV), they can positively or negatively influence subsequent infections and disease outcomes. Although there are some controversies, the majority of studies worldwide have described worse disease outcomes in HIV-, HBV- and HCV-infected individuals who were coinfected with HTLV-1, compared to those who were not coinfected. For instance, in HIV/HTLV-1-coinfected individuals from Salvador, Bahia, Brazil, rapid progression to acquired immune deficiency syndrome (AIDS) and death were observed^3^ ^,^ ^4^. In HBV/HTLV-1-coinfected individuals from Australia, the HBV viral load was increased when compared to their HBV-infected counterparts^5^ ^,^ ^6^. In HCV/HTLV-1-coinfected individuals from Japan, higher HCV viremia, a lower rate of sustained virologic response to α-interferon treatment, and an increased risk of chronic liver disease and hepatocellular carcinoma have been described^7^ ^-^ ^9^. In São Paulo, Brazil, increased HCV viremia was observed in patients with hepatitis C when coinfected with HIV and/or HTLV-1^10^. However, in Salvador, Bahia, patients coinfected with HIV and HTLV-1 were more likely to spontaneously clear the HCV than patients with HIV/HCV or HCV alone^11^. Conversely, coinfection with HTLV-2 has been shown to slow the progression to AIDS in HIV-infected individuals^12^ and to decrease HCV viral loads in people with hepatitis C^10^ ^,^ ^13^. Thus, the search for HTLV-1 and HTLV-2 in HIV-infected individuals, as well as in patients with HBV and HCV, has prognostic value.

Due to the high mortality and morbidity of HIV, HBV and HCV infections worldwide, together with the high transmission/dissemination capacity of these viruses, infection notification is compulsory in Brazil. According to the Brazilian Ministry of Health’s (MH) *Epidemiologic Bulletins of HIV/AIDS and Viral Hepatitis*, there were 926,742 cases of AIDS (1980 to 2018), 218,257 cases of hepatitis B (1998 to 2017), and 491,960 cases of hepatitis C (1999 to 2017); the majority of which were reported in the Southeast region of Brazil^14^ ^,^ ^15^.

In contrast, HTLV-1 and HTLV-2 are neglected infectious diseases in Brazil. There is no obligatory notification policy and these infections are not included on the list of neglected diseases; they are similarly mistreated in other parts of the world^16^. Thus, the real numbers of HTLV-1/-2-infected individuals in Brazil and elsewhere are unknown. Only considering published data, an estimated 800,000 HTLV-1-infected individuals were reported in Brazil in 2012^1^; this number might be underestimated^17^. In fact, another study conducted with blood donors from Brazil in 2005 estimated 2.5 million HTLV-1/2-infected individuals^18^. Thus, the notification of HTLV-1/-2 infections could solve the discrepant results and clarify the prevalence^16^ ^,^ ^17^.

Considering the data described above and the expertise of the Instituto Adolfo Lutz (IAL) of São Paulo from the studies on HTLV-1/2, two years ago, we decided to investigate the prevalence of HTLV-1 and HTLV-2 in patients with hepatitis B and C. The preliminary results concerning because included both HTLV-1 and HTLV-2: an overall prevalence of HTLV-1/2 of 1.3% in individuals with hepatitis B and 5.3% in individuals with hepatitis C, and an association with HIV in the HBV/HTLV-coinfected individuals^19^. Moreover, high HCV viral loads were detected in HCV/HIV- and/or HCV/HTLV-1-coinfected individuals^10^. Unfortunately, the low number of HBV/HTLV-coinfected individuals did not allow comparison between HBV-infected, HBV/HIV- and/or HBV/HTLV-coinfected individuals. At present, we decided to extend and confirm these preliminary data by conducting surveillance of HTLV-1, HTLV-2 and HIV in the blood samples of patients with viral hepatitis B and C in São Paulo. This would enhance the number of coinfected individuals to enable further HBV and HCV viral load and clearance analyses.

## METHODS

### Study population

The Instituto Adolfo Lutz (IAL), a public health laboratory in São Paulo, Brazil, and the reference laboratory for viral hepatitis, HIV and HTLV-1 and HTLV-2 infections, carried out the diagnosis and surveillance of these viruses. They also evaluated the epidemiological trends of the infections and coinfections. This institute has a central laboratory located in the city of São Paulo; analyses of high complexity are performed there. It also has 12 regional laboratories spread throughout the state of São Paulo. Patients who attended IAL came from different specialized health centers; this included STI/AIDS and viral hepatitis centers. A cross-sectional/transversal anonymous study was conducted using the data obtained from 1,984 plasma/serum samples. These were collected from 1,290 patients analyzed for HBV viral load (HBV group: 688 males, mean age of 48.2 years; 602 females, mean age of 45.8 years), and 694 patients analyzed for HCV viral load (HCV group: 391 males, mean age of 50.2 years; 303 females, mean age of 50.0 years). The collection occurred from June 2015 to June 2016 at the central IAL; the samples were stored at -20 °C.

**FIGURE 1:**
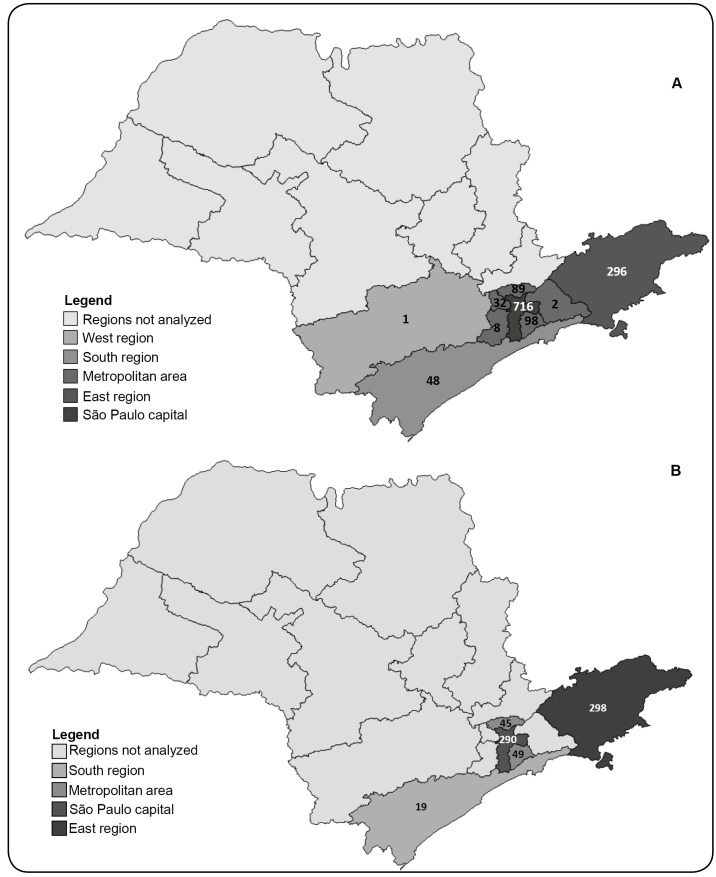
Maps of the state of São Paulo highlighting the regions from which the samples for hepatitis B (**A**) and hepatitis C (**B**) viral load measurement were collected. The number of samples from each region is depicted on the map.

The majority of samples were collected from the health centers in São Paulo city and its vicinity (metropolitan area) and in the cities in the eastern region of the state (up to 200 km from São Paulo city) (Figure 1).

The serological diagnoses of hepatitis B and C were performed at the specialized health centers where the patients were seen for clinical follow-up. These services used different immunoassays for the diagnoses, depending on availability (i.e., immunochromatographic, enzymatic or chemiluminescence assays). In some cases, HCV infections were confirmed using RNA quantification at IAL, using the Abbott Real-Time HCV assay (Abbott Molecular Inc., Illinois, USA).

### Laboratory Analyses

The samples were screened for HTLV-1/-2-specific antibodies at the HTLV Research Laboratory of the central IAL using enzyme immunoassay (EIA, Murex HTLV-I/II, Diasorin, UK). The reactions and interpretations of the results were performed according to the manufacturer’s instructions. The cut-off values and the gray zones were calculated; the samples considered reactive or inconclusive in the screening tests were further confirmed by Western blotting (WB) assay (HTLV BLOT 2.4, MP Biomedicals, Asia Pacific Pte Ltd.). For the WB confirmatory assay, the results were interpreted according to the stringent criteria provided by the manufacturer. Briefly, HTLV-1-positive serum samples were defined as the presence of gag (p19 with/without p24) and two env (GD21 and rgp46-I) bands. HTLV-2-positive samples were defined as reactive to gag (p24 with/without p19) and two env (GD21 and rgp46-II) bands. Samples showing antibodies to both gag (p19 and p24) and env (GD21) bands were considered HTLV-positive but untypeable. Any other patterns of bands were denoted as indeterminate.

In an attempt to confirm and/or to discriminate the samples with WB-inconclusive results (i.e., WB-indeterminate or HTLV-positive but untypeable), the samples were tested by line immunoassay (LIA; INNO-LIA HTLV-I/II, Fujirebio, Europe N.V., Belgium). The strips used in LIA contain antigens for validating, confirming and discriminating the results. For validation, the line marked by each sample was compared with the control line; a score ranging from +/- to +3 was assigned. The confirmatory antigens included gag p19 I/II, gag p24 I/II, env gp46 I/II, and env gp21 I/II. No bands, or the occurrence of a single band (gag p19 I/II, gag p24 I/II, or env gp46 I/II), denoted a negative result. The presence of one band (env gp21 I/II) or two bands (except env gp21 I/II) indicated indeterminate results, while two bands (env gp21 I/II and gag p19 I/II, gag p24 I/II, or env gp46 I/II) indicated HTLV positivity. Three discriminatory bands (gag p19-I, env gp46-I, and env gp46-II) were considered as follows: HTLV-1 positivity was indicated by the reactivity to gag p19-I and/or env gp46-I, while HTLV-2 positivity was considered when the samples showed env gp46-II or a higher intensity of the env gp46-II band than the gag p19-I and the env gp46-I bands. 

In addition, HIV infection was investigated by an EIA of high sensitivity and specificity, capable of detecting both HIV-1- and HIV-2-specific antibodies and the p24 antigen of HIV-1 (Murex HIV Ag/Ab Combination, Diasorin, UK). This assay allows the identification of early HIV infections. The reaction was carried out according to the manufacturer’s instructions; the results were defined as positive or negative for HIV infection.

### Statistical Analyses

The laboratory results and the patients’ demographic data were recorded and analyzed according to the Epi Info version 3.5.4 software (Atlanta, GA, USA). Differences in the number of males and females in each group were statistically evaluated using the chi-square test or Fisher’s exact test, as appropriate. GraphPad Prism software version 5.03 (San Diego, CA, USA) was employed for age comparisons among groups using the Mann-Whitney U test (two groups), the Kruskal-Wallis ANOVA test, and Dunn's Multiple Comparison Test (three or more groups). A *p*-value of ≤0.05 was considered significant. Logistic regression analysis was used to identify the factors associated with HTLV-1/2 infection by calculating odds ratios (ORs) and 95% confidence intervals (CIs).

### Ethical considerations

The study was approved by the Ethics Committee for Research at IAL (CTC#21I-2016) and received the MH protocol number: CAAE - 55837316.0.0000.0059. All procedures were performed in accordance with the principles established in the Declaration of Helsinki of 1975, as revised in 1983. The study was conducted anonymously.

## RESULTS

**FIGURE 2:**
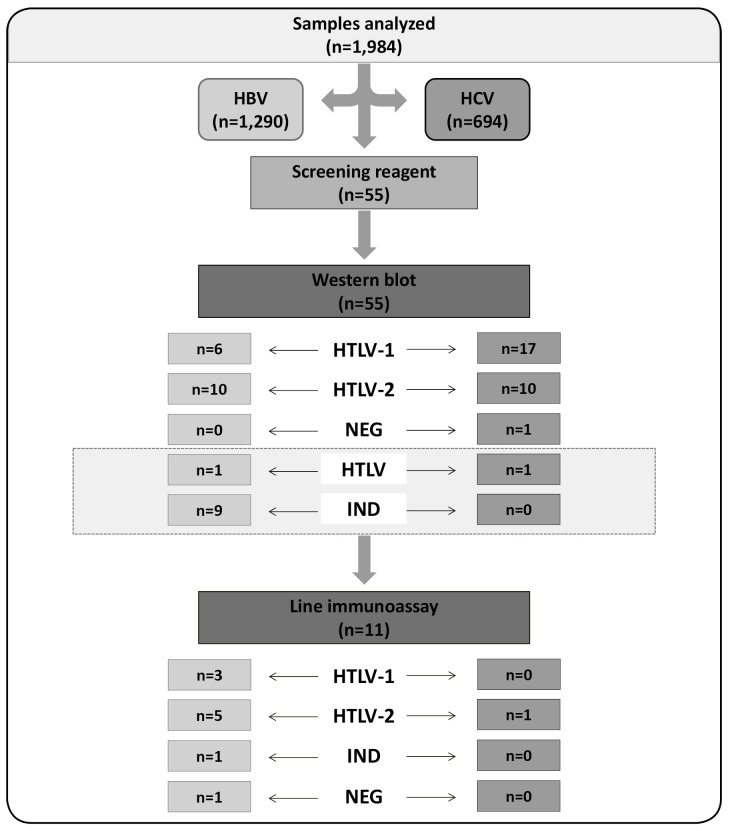
Diagram of HTLV serological results, with an emphasis on the indeterminate and HTLV-untypeable samples in the Western blot analyses and the final results of the line immune assays in blood samples from patients with hepatitis B and C in São Paulo. Serological assays and results are described in detail in the Methods section. **n:** number of samples; **HBV:** hepatitis B virus; **HCV:** hepatitis C virus; **HTLV-1:** human T-cell lymphotropic virus type 1; **HTLV-2:** human T-cell lymphotropic virus type 2; **HTLV:** human T-cell lymphotropic virus untypeable; **NEG:** negative sample; **IND:** indeterminate sample.

During the HTLV-1/-2 screening of the 1,984 plasma/serum samples, 55 reacted positively and were tested by WB; 43 had confirmed HTLV-1 or HTLV-2 positive results, 11 had WB-inconclusive results (nine WB-indeterminate, and two WB positive but HTLV untypeable results), and one had WB-negative result. After INNO-LIA analysis of the 11 WB-inconclusive samples, HTLV-1 or HTLV-2 was confirmed in nine samples, one sample was negative, and one remained indeterminate. The distribution of these samples, according to group (HBV or HCV), and the final HTLV results are presented in Figure 2.

Overall, 52 HTLV-positive samples were detected; 26 samples had confirmed HTLV-1 infections (HBV group: 9; HCV group: 17), and 26 samples had confirmed HTLV-2 infections (HBV group: 15; HCV group: 11). The HTLV-1/-2 and HIV serological results in each group (HBV and HCV) are presented in Table 1. Briefly, the overall prevalence of HTLV among the 1,290 HBV-infected individuals was 1.9% (0.7% HTLV-1; 1.2% HTLV-2); the corresponding prevalence among the 694 HCV-infected individuals was 4.0% (2.4% HTLV-1; 1.6% HTLV-2). Of the 24 HBV/HTLV-coinfected patients, 58.3% were HIV positive, compared to 8.1% of their HBV-infected counterparts (OR: 15.48 [95% CI: 6.28 - 38.59]). Of 28 the HCV/HTLV-coinfected patients, 53.6% were HIV positive, compared to 12.4% of their HCV-infected counterparts (OR: 7.78 [95% CI: 3.36-18.08]) (Table 1).

**TABLE 1:** Serological HTLV-1/-2 and HIV results in blood samples from patients infected with HBV and HCV in São Paulo, Brazil

Groups		HBV	HCV
		(n=1,290)	(n=694)
		N (%)	N (%)
**HTLV-1/2** ^a^	HTLV-1	9 (0.70)	17 (2.4)
	HTLV-2	15 (1.2)	11 (1.6)
	**Total**	**24 (1.9)**	**28 (4.0)**
**HIV** ^b^	HIV	105 (8.1)	86 (12.4)
	HIV/HTLV-1	3 (0.2)	5 (0.7)
	HIV/HTLV-2	11 (0.9)	10 (1.4)
	**Total**	**119 (9.2)**	**101 (14.5)**

**n:** number of analyzed samples; ^a^ **:** results obtained by enzyme immunoassay (Murex HTLV-I/II, Diasorin, UK) and confirmed by Western blot (HTLV BLOT 2.4, MP Biomedicals, Asia Pacific Pte Ltd.) and/or line immunoassay (INNO-LIA HTLV-I/II, Fujirebio, Europe N. V, Belgium); ^b^ **:** results obtained by enzyme immunoassay (Murex HIV Ag/Ab Combination, Diasorin, UK); **HTLV-1/2:** human T-cell lymphotropic virus type 1 and 2; **HIV:** human immunodeficiency virus.

Three regions of the state of São Paulo that sent samples to the IAL accounted for the positive HTLV-1/-2 results. In the HBV-infected individuals, 2.4% came from the east region, 1.8% came from the metropolitan area of São Paulo city, and 1.8% came from São Paulo city. In the HCV-infected individuals, 4.7%, 3.2%, and 3.8% came from the regions described for HBV, respectively.

**FIGURE 3:**
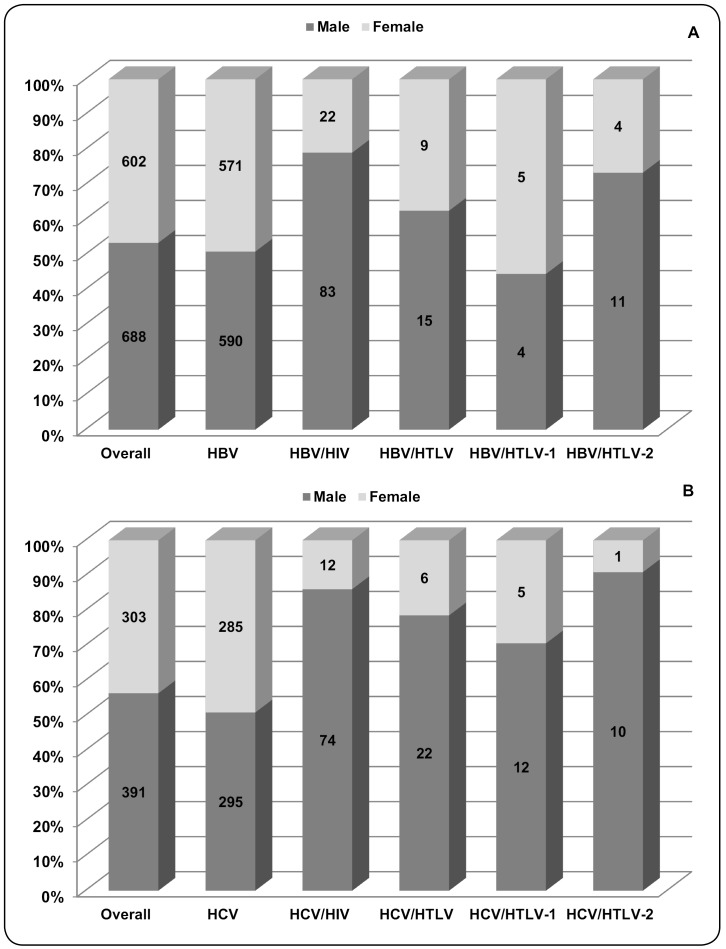
The number and percentage of patients with hepatitis B (**A**) and hepatitis C (**B**) whose blood samples were analyzed for HTLV-1/2- and HIV-coinfections, according to serological results and sex. Serological assays and results of HTLV-1, HTLV-2, and HIV are described in detail in the Methods section. **HBV:** hepatitis B virus; **HBV/HIV:** hepatitis B virus and human immunodeficiency virus coinfection; **HBV/HTLV:** hepatitis B virus and human T-cell lymphotropic virus coinfection; **HBV/HTLV-1:** hepatitis B virus and human T-cell lymphotropic virus type 1 coinfection; **HBV/HTLV-2:** hepatitis B virus and human T-cell lymphotropic virus type 2 coinfection; **HCV:** hepatitis C virus; **HCV/HIV:** hepatitis C virus and human immunodeficiency virus coinfection; **HCV/HTLV:** hepatitis C virus and human T-cell lymphotropic virus coinfection; **HCV/HTLV-1:** hepatitis C virus and human T-cell lymphotropic virus type 1 coinfection; **HCV/HTLV-2:** hepatitis C virus and human T-cell lymphotropic virus type 2 coinfection.

According to sex, there were more males in both groups of this study (HBV: 53.3%; HCV: 56.3%), with a relationship between males and females close to 1.0 in the HBV-infected and HCV-infected patients (Figure 3). However, when sex was compared among groups (HIV- and/or HTLV-coinfected), the male to female ratios increased in the following coinfection groups: 3.8 in the HBV/HIV group (*p*<0.0001), 1.7 in the HBV/HTLV group (*p*=0.257), 6.2 in the HCV/HIV group (*p*<0.0001), and 3.7 in the HCV/HTLV group (*p*=0.004). The number of individuals in each group and the percentage of patients according to sex are depicted in Figure 3. In addition, these results suggest that men were more likely to have an HIV infection concurrently with hepatitis B or C (OR: 3.65 [95% CI: 2.27-3.65] and OR: 5.91 [95% CI: 3.19-11.12], respectively), and an HTLV coinfection with hepatitis C (OR: 2.95 [95% CI: 1.12-8.23]).

**FIGURE 4:**
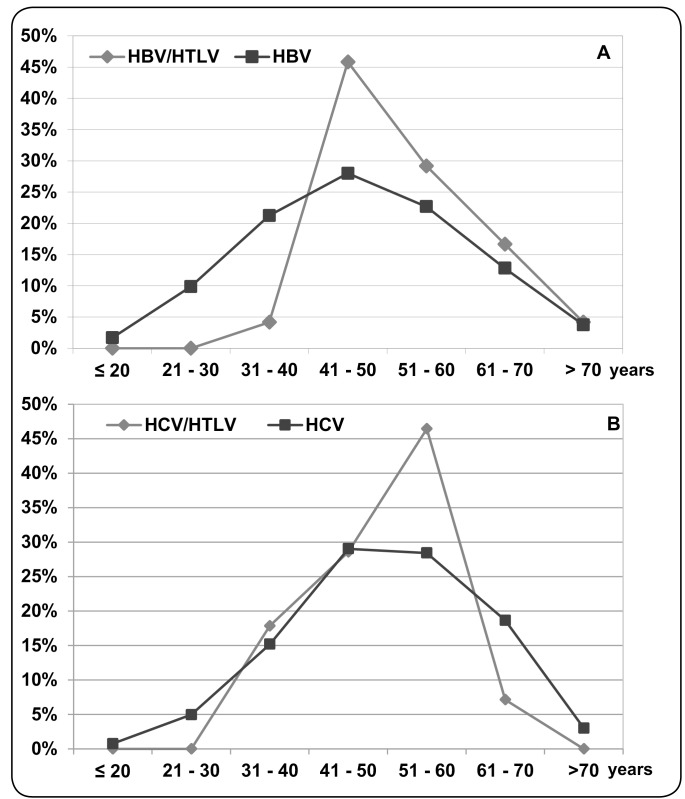
Percentages of HBV-infected and HBV/HTLV-1/2-coinfected patients (**A**) and HCV-infected and HCV/HTLV-1/2-coinfected patients (**B**), according to age group. **HBV:** hepatitis B virus; **HBV/HTLV:** hepatitis B virus and human T-cell lymphotropic virus coinfection; **HCV:** hepatitis C virus; **HCV/HTLV:** hepatitis C virus and human T-cell lymphotropic virus coinfection.

Considering the age of the patients, the median age differed between the HBV-infected and HBV/HTLV-coinfected patients (47.0 vs. 50.5 years old, *p*=0.0434). The majority of coinfected individuals had ages ranging from 41 to 60 years old, however, most of them were 41 to 50 years (Figure 4A). Among the patients with hepatitis C, the median age of the HCV-infected and HCV/HTLV-coinfected individuals was 51.0 years old for both. Although the majority of the HCV/HTLV-coinfected patients were between the ages of 41 and 60 years old, most of them were 51 to 60 years old (*p*=0.046); this was ten years above the median age of HBV/HTLV-coinfected patients (Figure 4B).

## DISCUSSION

The current study presents the results of the surveillance of HTLV-1, HTLV-2, and HIV in HBV- and HCV-infected patients by analyzing the serum/plasma samples for HBV and HCV viral load measurements at the central IAL. The results obtained showed a high prevalence of HTLV-1/-2: 1.9% and 4.0% in samples from HBV- and HCV-infected individuals, respectively. These results are similar to the percentages reported in a preliminary study conducted in São Paulo one year before^19^. Curiously, no significant differences were found in the HTLV-1/2 positive results according to the geographic regions or the services that sent the samples to the IAL for analysis. The same services and regions accounted for the positive HTLV-1/2 results in both the HCV- and HBV-infected individuals. The percentages of HTLV-1/2 detected herein were of concern as they were similar to those detected in an HTLV-endemic area (1.8%)^20^ and in populations with HIV/AIDS from São Paulo (3.1% to 4.2%)^21^ ^-^ ^23^.

Indeed, the present study showed the difficulty of confirming and discriminating HTLV-1 and HTLV-2 using the WB assay (HTLV Blot 2.4). This finding is also concerning as HTLV-1 and HTLV-2 seem to influence hepatic disease outcomes differently. Once more, the LIA was identified as the best assay for confirming HTLV-1/2 infections in such patients; the same finding was previously detected for patients with HIV/AIDS in São Paulo^23^ ^-^ ^24^. Curiously, in this study, the majority of samples with WB-inconclusive results were from HBV-infected individuals, and they were confirmed as HTLV-2-infected with the LIA. Corroborating these data, difficulties in confirming the infection status of (mostly) HTLV-2 using the WB assay had been previously described by us since the 1990s, when the infections were confirmed through molecular assays (PCR, RFLP-PCR, and qPCR)^25^ ^-^ ^29^.

Interestingly, although the WB assays have improved over time, the number of WB-inconclusive results in Brazilian blood samples currently remains. In addition, they include samples from high-risk populations (HIV-, HBV-, and HCV-infected individuals), and samples of HTLV isolated from the outpatient clinics in HTLV-1 endemic areas^24^ ^,^ ^30^. Thus, the low sensitivity of the WB assay is persistent with Brazilian blood samples; it is not limited to at-risk individuals in Brazil. The high cost of the LIA was not conducive its use in routine testing at public health laboratories in Brazil; however, this has changed recently as the costs of WB and LIA have become quite similar in the country.

Another important finding that emerged from the present study was the increased risk of HIV infection in HBV/HTLV- (OR: 15.48) and HCV/HTLV-coinfected patients (OR: 7.73). In fact, when the blood samples from HBV- and HCV-infected individuals were tested for HIV infection, 9.2% and 14.5% were positive, respectively. This was somewhat above the percentages (7.9% and 8.9%, respectively), in HBV- and HCV-infected individuals reported by the southeast region to the Brazilian MH (2018)^15^. These percentages, however, contrast with the high percentages of HIV infection detected in the HBV/HTLV- and HCV/HTLV-coinfected individuals in this study (58.3% and 53.6%, respectively). These percentages led us to suppose that these individuals could acquire HBV and HCV, as well as HTLV-1/2 and HIV, at the same time, via the same route of transmission. At present, the main routes of HTLV-1/2 transmission in Brazil are the vertical route, through breastfeeding newborns for longer than six months, and the sexual route, through unprotected sexual intercourse^31^ ^-^ ^33^. The parenteral routes (e.g., blood transmission, solid organ and cell transplantation, and intravenous drug use [IDU]), have been less frequent since some public health policies were adopted in Brazil. For instance, HTLV-1/2 serology has been mandatory in blood banks in Brazil since 1993^34^. Further, serology for donors and recipients of solid organs and hematopoietic cell transplants has been required since 2009^35^. The harm reduction program, adopted by the Brazilian MH for HIV, provides sterile needles and syringes to intravenous drug users^36^. These initiatives contributed to a reduction in retrovirus transmission. However, HTLV serology is not compulsory for pregnant women in antenatal care; this is a contradiction, as since 2009, the MH recommends that mothers infected with HTLV-1/2 avoid breastfeeding^37^.

In the present study, although we did not know the routes of viral transmission or the temporal order of the infections, we could speculate that transmissions occurred mostly through parenteral routes. Prior to laboratory testing being commercially available, and HIV and HBV serology (1988) and HTLV and HCV serology (1993) became mandatory in blood banks throughout Brazil^34^ ^,^ ^38^. We suspect that infection occurred mostly when IDU was more frequent in this country, as previously described^21^ ^,^ ^25^ ^,^ ^26^ ^,^ ^36^. Corroborating this hypothesis, there was a predominance of HBV/HTLV- and HCV/HTLV-coinfected patients who were older males, with significant differences compared to their HBV- and HCV-infected counterparts. Conversely, these findings could not exclude the possible acquisition of such viruses sequentially through the sexual route as there was a 10-year difference in the common ages of HBV/HTLV- (41 to 50 years) and HCV/HTLV-coinfected (51 to 60 years) individuals. Additionally, HBV is typically considered a sexually transmitted infection, while HCV is predominantly acquired parenterally^15^.

Another important point that emerged from the present study is that, while both HTLV-1 and HTLV-2 were detected in HBV- and HCV-infected patients, there were more HTLV-2 cases in the HBV-infected individuals, and more HTLV-1 cases in the HCV-infected individuals. Whether these findings were beneficial or not, regarding the hepatitis B and hepatitis C outcomes, remains to be determined.

This study had one major limitation. The results obtained could not allow us to determine the precise prevalence of HTLV-1/2 in this state for the several reasons. First, it was a cross-sectional/transversal study that employed samples from patients who were initiating treatment for hepatitis B and C or who were in follow-up. Second, the samples came mostly from the east region of the state of São Paulo; as such, they may not be generalizable to the rest of the state.

In conclusion, HTLV-1 and HTLV-2 were confirmed to be prevalent in individuals with viral hepatitis B and C in São Paulo. We are now investigating the HBV and HCV viral loads and clearance data from these patients to enhance the information concerning this matter. This will better assist physicians in performing accurate patient follow-ups. Confirming the virological prognostic values of these infections may help convince the Brazilian MH to add HTLV-1/2 serology to the battery of tests used in the follow-up of patients with viral hepatitis B and C.
